# Supplementary material for: Generation and Characterization of a Novel Angelman Syndrome Mouse Model with a Full Deletion of the Ube3a Gene
Source: Cells. 2022 Sep 9;11(18):2815. doi: 10.3390/cells11182815 (PMC9496699; doi:10.3390/cells11182815)
Supplement: Supplementary file 1 [file cells-11-02815-s001.zip › Supplementary 1. Rotarod weight.pdf]

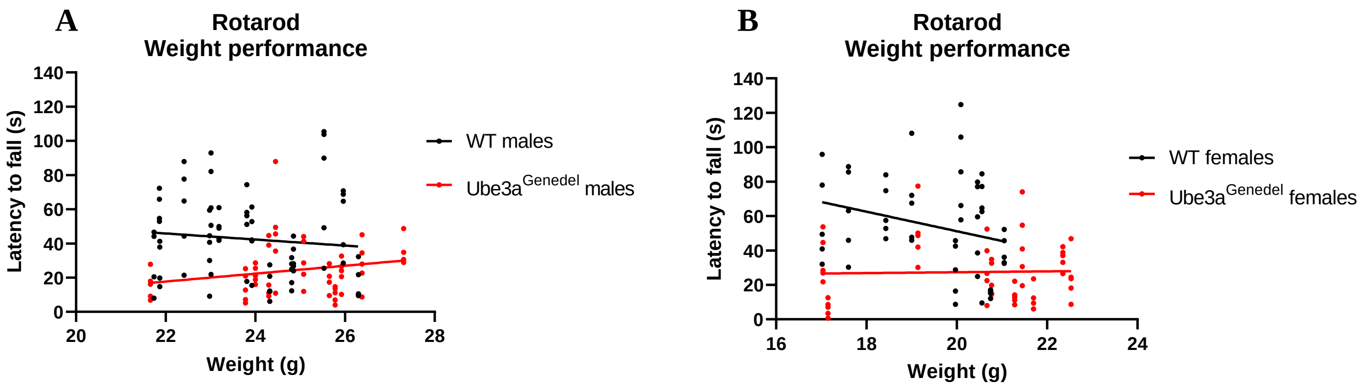

Supplementary Figure S1. Weight and latency to fall in rotarod. **A-B** Weight of the animals plotted against the latency to fall, pooled for all days, linear model fit main weight effect p=0.68, mean ± SD.
