# Supplementary material for: Generation and Characterization of a Novel Angelman Syndrome Mouse Model with a Full Deletion of the Ube3a Gene
Source: Cells. 2022 Sep 9;11(18):2815. doi: 10.3390/cells11182815 (PMC9496699; doi:10.3390/cells11182815)
Supplement: Supplementary file 1 [file cells-11-02815-s001.zip › Supplementary 2. Intellicage.pdf]

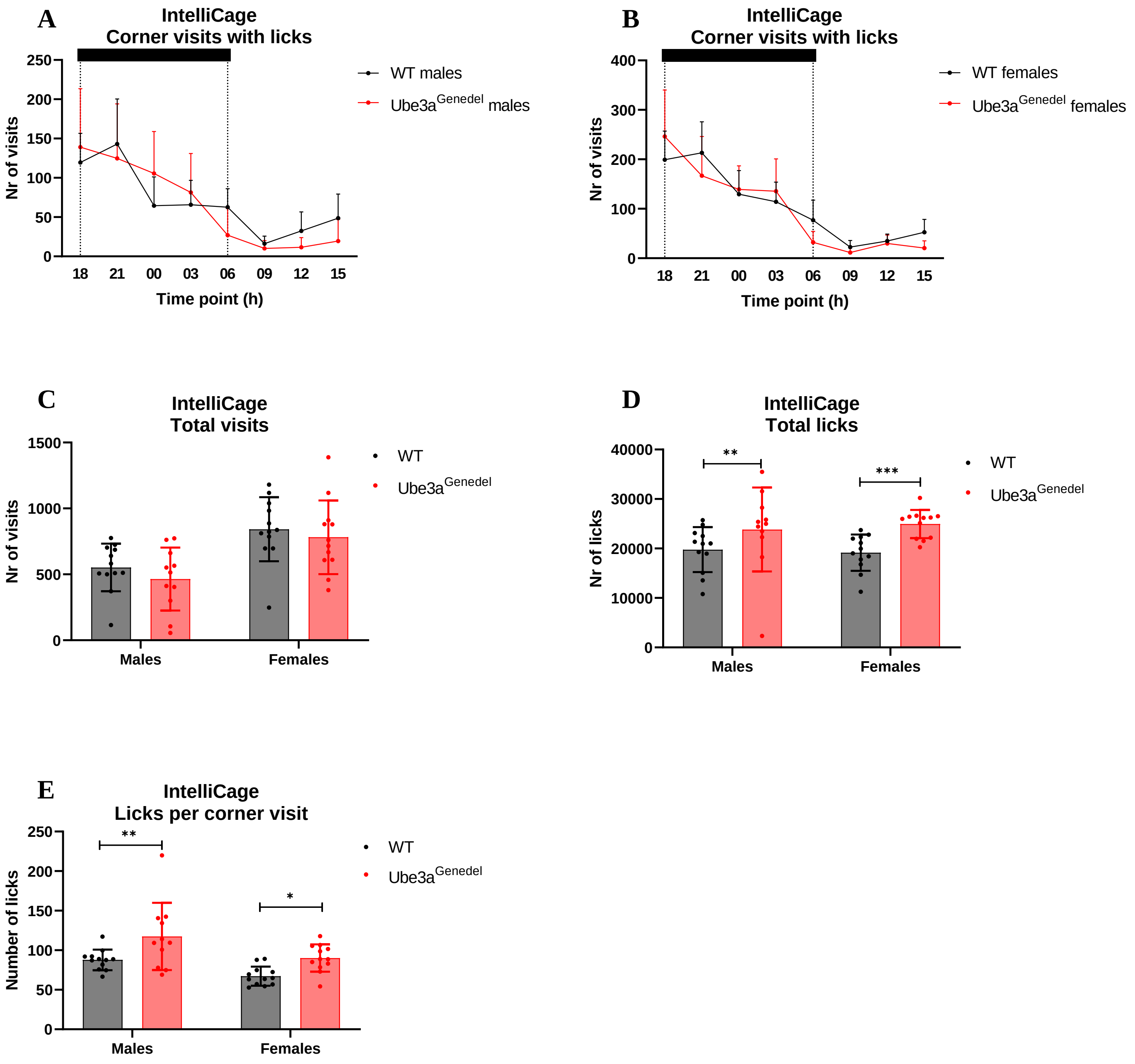

Supplementary Figure S2. IntelliCage results licks and corner visits. A-B Circadian activity in corner visits not differing between Ube3agenedel and WT mice. c Overall corner visits does not differ over a 7-day period. D Ube3agenedel mice exhibit an increase in lick number over the entire period of 7 days. E Ube3agenedel mice displayed more licks per visit than WT controls. Two-way ANOVA with dependent measurements, genotype main effect, $p<0.001$, depicted with mean ± SD. Significant effects of genotype are indicated as *$p < 0.05$, **$p < 0.01$, and ***$p < 0.001$, ****$p < 0.0001$ for genotype significance
